# *Polygoni Multiflori Radix Praeparata* Ethanol Extract Exerts a Protective Effect Against High-Fat Diet Induced Non-Alcoholic Fatty Liver Disease in Mice by Remodeling Intestinal Microbial Structure and Maintaining Metabolic Homeostasis of Bile Acids

**DOI:** 10.3389/fphar.2021.734670

**Published:** 2021-11-15

**Authors:** Xuyang Dai, Linfeng He, Naihua Hu, Chaocheng Guo, Mengting Zhou, Xingtao Zhao, Cheng Wang, Lihong Gong, Cheng Ma, Xinyan Xue, Yunxia Li

**Affiliations:** School of Pharmacy, Key Laboratory of Standardization for Chinese Herbal Medicine, Ministry of Education, National Key Laboratory Breeding Base of Systematic Research, Development and Utilization of Chinese Medicine Resources, Chengdu University of Traditional Chinese Medicine, Chengdu, China

**Keywords:** NAFLD, high-fat diet, intestinal barrier, intestinal microbial, bile acids

## Abstract

In the prescription of Traditional Chinese Medicine for lipid metabolism, *Polygoni Multiflori Radix Preparata* (ZhiHeShouWu, RPMP) was widely used. In recent years, RPMP ethanol extract has been reported for the treatment of non-alcoholic fatty liver disease (NAFLD). However, the role of RPMP ethanol extract in the treatment of NAFLD has not been fully elucidated. Therefore, we examined the optimal therapeutic dose of RPMP ethanol extracts. Afterward, a mouse model of non-alcoholic fatty liver induced by a high-fat diet (HFD) was treated with RPMP ethanol extract to further evaluate the mechanism of action of RPMP ethanol extract treatment. And the serum lipid metabolism indexes and liver function indexes showed that the RPMP ethanol extract in the 1.35 g/kg dose group exhibited better therapeutic effects than the 2.70 g/kg dose group. Meanwhile, RPMP ethanol extract can regulate the biochemical indicators of serum and liver to normal levels, and effectively reduce liver steatosis and lipid deposition. RPMP ethanol extract treatment restored HFD-induced disruption of the compositional structure of the intestinal microbial (IM) and bile acids (BAs) pools. And restore the reduced expression of intestinal barrier-related genes caused by HFD administration, which also effectively regulates the expression of genes related to the metabolism of BAs in mice. Thus, RPMP ethanol extract can effectively improve the abnormal lipid metabolism and hepatic lipid accumulation caused by HFD, which may be related to the regulation of IM composition, maintenance of intestinal barrier function, and normal cholesterol metabolism in the body.

## Introduction

Non-alcoholic fatty liver disease (NAFLD) is a general term that used to describe a series of abnormal hepatic lesions, the disease progression of which beginning from simple steatosis to non-alcoholic steatohepatitis (NASH) characterized by hepatocyte degeneration, necrosis, and accompanied by varying degrees of inflammatory cell infiltration. The deterioration of NAFLD can lead to cirrhosis and liver cancer. Changes in people’s lifestyles and dietary modes as a result of industrialization development, NAFLD has become the most common liver disease, the proportion of people who suffered from NAFLD worldwide which was rising all the time was between 20 and 30%, and 10–20% of them will develop into NASH (Yamamura et al., 2021).

A variety of biological processes, such as abnormal lipid metabolism and insulin resistance, have contributed to the occurrence and development of NAFLD, of which intestinal microbial (IM) imbalance has attracted much attention in the past decade due to the unique connection between the anatomical location of the intestine and liver and the emergence of new microbiome sequencing methods. There was a significant increase in the bacterial abundance of Bacteroides and Prevotella in NASH patients; In the meantime, the proportion of Bacteroides in NASH patients was inversely correlated to that of Prevotella (Yatsunenko et al., 2012; Suk and Kim, 2019; Boursier et al., 2016). Although there were some contradictory phenomena, it was enough to realize that IM has corresponding changes in the development of NAFLD. Furthermore, it was found that showed an increase in body fat content and insulin resistance after transplanting IM from the traditional feeding mice into the germ-free (GF) mice (Bäckhed et al., 2004). And, when GF mice raised on a low-fat diet exhibited an obesity-associated metabolic phenotype when subjected to IM by adult obese twins (Ridaura et al., 2013). While there was no obesity induced by the western diet in GF mice under the intervention of high fat and high sugar diet (Bäckhed et al., 2007). Therefore, we can’t ignore the role of IM in the development of NAFLD. In addition, endogenous molecular bile acids (BAs), which are produced by hepatocytes in charge of emulsification and cleaning, are regulated by liver biosynthesis and IM in the body. And research shows that BAs sequestration can prevent the progression of NASH (Takahashi et al., 2020). What’s more, the evidence for the correlation between BAs and NAFLD has increased (Gupta et al., 2020; Zeng et al., 2020).

NAFLD is so haunting us, but it is still a lack of effective drugs to control the development of NAFLD. *Polygoni Multiflori Radix*—the dry root tuber of *Polygonum multiflorum Thunb* (Polygonaceae, HeShouWu). And *Polygoni Multiflori Radix Praeparata* (ZhiHeShouWu, RPMP) is obtained by processing *Polygoni Multiflori Radix* in black bean juice, which was used to regulate lipid metabolism in clinical frequently. Modern pharmacological studies also claimed that RPMP has a protective effect on the invasion of the fatty liver (Li et al., 2018). These studies provided us strong support to further explore the regulation of RPMP on the progression of NAFLD. At the same time, the group used high-performance liquid chromatography (HPLC) to quantify the 50% ethanol extract and the aqueous extract of RPMP respectively, and the results showed that the ethanol extract of RPMP was superior to the aqueous extract in terms of the content of the six components examined [2,3,5,4′-tetrahydroxystilbene-2-O-β-d-glucoside (TSG), emodin, physcion, catechin, gallic acid ester, emodin glycoside]. And it has shown positive therapeutic effects on animal models of NAFLD (Yu et al., 2020). Therefore, we attempted to investigate the role of RPMP in the occurrence and progression of NAFLD from the perspectives of the connection between IM and BAs.

## Materials and Methods

### Drugs and Reagents

*Radix Polygoni Multiflori Praeparata* (No. 1901026) was purchased from Sichuan Neautus Traditional Chinese Medicine Co., Ltd., (Chengdu, China), and authenticated by professor Pei Jin, department of pharmacognosy, Chengdu University of Traditional Chinese Medicine, and the samples were deposited at the Herbal Medicine Museum. A high-fat diet (No. 2019022301) was provided by Biotech-HD Co., Ltd., (Beijing, China), and the diet composition was reflected in Table 1. HPLC grade acetonitrile, methanol, and formic acid were obtained from Merck Chemicals (Shanghai, China), Wokai Chemical Technology Co., Ltd. (Shanghai, China), and TCI Chemical Industry Development Co., Ltd. (Shanghai, China), respectively. The kit for detecting the levels of total cholesterol (TC) (No. 20200114), triglyceride (TG) (No. 20200114), low-density lipoprotein cholesterol (LDL-C) (No. 20200114), high-density lipoprotein cholesterol (HDL-C) (No. 20200114), Alanine transaminase (ALT) (No. 20200114), and Aspartate transaminase (AST) (No. 20200114) in serum and liver tissue were provided by Nanjing Jiancheng Bioengineering Institute (Nanjing, China). BCA Protein Quantification Kit (No. A00445) was obtained from Multi Sciences Biotech Co., Ltd. TRIzol reagents (No. 175702) used to extract total RNA from the liver and ileum tissue was purchased from Ambion Life Technologies (Carlsbad, CA, United States). 5X All-In-one MasterMix (No. 65170013) and Eva Green 2X RT-qPCR MasterMix-Low RoX (No. 65170013) were purchased from Applied Biological Materials Inc. (Richomnd, BC, Canada). PCR primer sequences were synthesized by TSINGKE Biological Technology (Chengdu, China). Other reagents used in this experiment were provided by Kelong Chemical Reagent Factory (Chengdu, China).

**TABLE 1:** Compositions of animal diets.

	Normal diet (ND)		High fat diet (HFD)	
Ingredients	Mass percentage (gm%)	Percent of calories (kcal%)	Mass percentage (gm%)	Percent of calories (kcal%)
Protein	21.1	19.7	24.2	19.8
Carbohydrate	60.6	70.3	42.1	35.2
Fat	4.5	10.0	25.4	45.0
Crude fiber	7.7	—	5.8	—
Calcium	1.8	—	1.7	—
Phosphorus	1.2	—	1.1	—

### Preparation and Quality Analysis of Ethanol Extract

After precise weighing and sieving, 100 g of RPMP crude powder was placed in a round bottom flask with ten times the amount of 50% ethanol and soaked for 30 min, heat reflux for 60 min, filter. Then add eight times the amount of 50% ethanol, heat reflux for 60 min, filter, and combine the filtrate and the filtrate was concentrated by rotary evaporation at 45°C to contain 1 g of raw drug per ml and stored at 4°C.

Quantitative analysis of RPMP ethanol extract by HPLC based on a ZORBAX C18 analytical column (4.6 mm × 250 mm, 5 μm) with a pre-column ZORBAX C18 (4.6 mm × 12.5 mm, 5 μm). Refer to our previously established method (Yu et al., 2020). The gradient elution was carried out with 0.1% formic acid (A) and acetonitrile (B) as mobile phases at a column temperature of 30°C, a detection wavelength of 275 nm, and a flow rate of 1 ml/min according to the following mobile phase composition: (0–5 min) 5–10% B, (5–30 min) 10–22% B, (30–38 min) 22–25% B, (38–48 min) 25–32% B, (48–55 min) 32–45% B, (55–65 min) 45–85% B, (65–70 min) 85–95% B, (70–72 min) 95–95% B.

### Animals and Experimental Groups

All experimental procedures were conducted in accordance with animal experimental protocols and guidelines approved by the Animal Ethics Committee of the Chengdu University of Traditional Chinese Medicine (Approval No. AEC-201611). 70 male Kunming mice (km) from Chengdu Dossy Experimental Animal Co., LTD. [Chengdu, China, No. SCXK (Chuan) 014–028] were housed for 1 week under standard environment (temperature of 25 ± 2°C, the humidity of 50 ± 5%, and a 12 h light/12 h dark cycle) with free access to sterile water and normal chow. All efforts were made to minimize animal suffering. And at the end of the adaptation, the following experiments were performed:

1) To determine the threshold for the therapeutic dose of RPMP ethanol extract, a dose conversion for mice was performed based on the guideline dosage of 9 g of RPMP for humans provided in the Chinese Pharmacopoeia (Mouse dose = Human dose/60 kg × 9.01). The km mice were randomly divided into five groups (*n* = 6/group) and treated with normal diet (ND) (CON), HFD (MOD), HFD and different doses of RPMP ethanol extract [0.68 g/kg (0.68 g/kg RPMP), 1.35 g/kg (1.35 g/kg RPMP), 2.70 g/kg (2.70 g/kg RPMP)] for 3 weeks. At the end of week 3, blood samples were collected from each group of mice.

2) To further investigate the role played by RPMP ethanol extract in the development of NAFLD, km mice were randomly divided into two groups for 3 weeks of ND (CON) (*n* = 8) and HFD administration (*n* = 32). And then the HFD-fed mice were randomly divided into HFD (MOD), HFD, and different doses of RPMP ethanol extract [0.34 g/kg (0.34 g/kg RPMP), 0.68 g/kg (0.68 g/kg RPMP), 1.35/kg (1.35 g/kg RPMP)] (*n* = 8/group) groups for 5 weeks of treatment, and continued to be supplemented with ND and HFD treatment during this process. Fecal samples were collected at week 8. And at the end of the experiment, blood, liver tissue, epididymal adipose tissue, and ileal tissue were collected.

Mice were treated with RPMP ethanol extract at 9:00 am and 18:00 pm daily. The body weight of mice was measured weekly and treatment volumes were adjusted. The volume of the extract administered was calculated according to the following formula. In order to reduce the interference of treatment volume, double distilled water was used to dilute RPMP ethanol extract with the initial concentration of 1 g/ml to 0.0625 g/ml (0.34 g/kg RPMP), 0.125 g/ml (0.68 g/kg RPMP), and 0.25 g/ml (1.35/ kg RPMP) for different dose groups. Also, during the RPMP ethanol extract intervention, the CON and MOD groups were given the same volume of double distilled water according to body weight.

$$\text{The}\;\;\text{administration}\;\text{volume}\;\text{of}\;\text{each}\;\text{mouse}\left(\text{ml}\right)=\frac{\text{Body}\;\text{weight}\;\text{of}\;\text{mice}\left(\text{kg}\right)\times \text{Administration}\;\text{dose}\left(\text{g}/\text{kg}\right)}{\text{The}\;\text{concentration}\;\text{of}\;\text{the}\;\text{extract}\left(\text{g}/\text{ml}\right)}$$

### Fasting Glucose Level Test

After overnight fasting, fasting blood glucose values were measured in mice using a blood glucose meter (ACCU-CHEK Performa, Performa).

### Sample Collection

Blood samples. The blood of mice was obtained through the orbital vein plexus. The collected blood samples were allowed to stand at room temperature for 1 h, then centrifuged at 2,500 r/min for 15 min at 4°C. The supernatants were aspirated and stored in separate packs.

Tissue sample. The sacrifice of mice was accomplished by cervical dislocation. Abdominal dissection was performed to extract the liver, epididymal fat, and ileal tissue. The blood was removed by rinsing in ice-cold saline, and filter paper was swabbed dry before weighing and recording the weight of liver and epididymal adipose tissue. And the tissue samples were stored in 4% paraformaldehyde or liquid nitrogen.

Fecal samples. The mice were fixed in an ultra-clean worktable, their tails were lifted, and their abdomens were gently stroked with fingers to stimulate defecation. Fresh feces were collected in sterile lyophilized tubes, quickly placed in liquid nitrogen, and transferred to a refrigerator (−80°C) for storage.

### Biochemical Indexes

Serum TC, TG, LDL-C, HDL-C, ALT, and AST levels were measured using the above-preserved blood samples and according to the kit instructions. Take 0.1–1 g liver tissue was homogenized by adding 0.86% cold saline into pipette in the proportion of homogenate medium: tissue weight (1:9). The prepared 10% homogenate was centrifuged at 2,000 r/min for 10 min, and the supernatant was reserved. Then, the liver levels of TC, TG, LDL-C, HDL-C, ALT, and AST were also measured according to the kit instructions.

### Histopathology

The liver and colon samples were fixed with 4% paraformaldehyde, embedded in paraffin and sectioned (5 μm), stained with hematoxylin-eosin (HE). And lipid accumulation in the liver was observed by staining frozen sections of the liver with an oil red O stain. The frozen sections were fixed in fixative for 15 min, washed in PBS, dried and immersed in oil red O staining solution for 10 min, and then background fractionated with 60% isopropyl alcohol, and finally sealed with glycerol gelatin sealer after hematoxylin re-staining. And then observed under a light microscope (CX21FS1, OLMPUS, Japan) and the pathological changes of the liver and colon were photographed under a microscopic imaging system (DM1000, Leica, Germany).

### Intestinal Microbial Sequencing

The genomic DNA of fecal samples was extracted by using QIAamp Fast DNA Stool Mini Kit (Qiagen, Germany), then detected by electrophoresis with 0.8% agarose gel. The V4 region of the bacterial 16S rDNA gene was amplified by polymerase chain reaction (PCR) with 515F–806R primer set (515F:5′-GTGYCAGCMGCCGCGGTAA-3′,806R:5′-GGACTACHVGGGTWTCTAAT-3′), three replicates per sample, each PCR reaction was terminated in the linear amplification period. PCR product of the same sample was mixed and detected by electrophoresis with 2% agarose gel, then purified by Qiagen Gel Extraction Kit (Qiagen, Germany). PCR products quality were evaluated on Qubit@ 2.0 Fluorometer (Thermo Scientific, United States), and then mixed in equidensity ratios. Sequencing libraries were prepared using the TruSeq DNA PCR-Free Sample Prep Kit (Illumina, No. FC-121-3001/3003). The library was sequenced on an Illumina Hiseq 2500 platform and paired-end reads were generated.

QIIME pipeline (qiime2) was used to process and analyze the raw data. Final Clean Reads were produced after the removal of chimera sequences in the tags, which were detected by the gold database (microbiomeutil-r20110519). Operational taxonomic units were clustered at 97% similarity, and sequences were taxonomically assigned against the SILVA database (Release_132). The raw data of libraries generated during this study is publicly available at the Sequence Read Archive (SRA) portal of NCBI (https://www.ncbi.nlm.nih.gov/sra/) under accession number PRJNA769303.

### Determination of Bile Acids in Feces of Mice

The quantitative analysis of mouse fecal BAs was done by liquid chromatography-mass spectrometry (LC-MS). Briefly, 10 mg of mouse feces was accurately weighed and suspended in 1 ml of methanol solution (−20°C). Ultrasonication at room temperature for 30 min, centrifugation at 12,000 rpm/min for 10 min at 4°C using a frozen centrifuge (H1850R, Cence). Then, 300 μl of the supernatant was filtered into the assay bottle with the assistance of a 0.22 μm membrane. Based on an ACQUITY UPLC BEH C18 column (2.1 mm × 100 mm, 1.7 μm, Waters), the elution was performed at a column temperature of 40°C and a flow rate of 0.25 ml/min using 0.01% formic acid (A) and acetonitrile (B) as mobile phases according to the following gradient of mobile phase composition: (0–4 min)25% B, (4–9 min)25–30% B, (9–14 min)30–36% B, (14–18 min)36–38% B, (18–24 min) 38–50% B, (24–32 min) 50–75% B, (32–35 min) 75–100% B, (35–38 min) 100–25% B. And scanned using multiple reaction monitoring (MRM).

### Quantitative RT-qPCR

Total RNA was extracted from liver and ileal tissues by using TRIZOL reagent and resuspended in 50 μl of enzyme-free water. RNA purity and integrity verified by optical density 260/280 values measured by Nucleic Acid/Protein Analyzer and Type 1 nucleic acid dye on 1% agarose gel. Reverse transcription for cDNA synthesis with 5X All-In-One MasterMix. Set reaction conditions according to the manufacturer’s instructions to complete the RT-qPCR reaction. Then, the relative trends of the genes were analyzed according to the amplification curves and the calculation method based on 2^−∆∆CT^. Primer sequences were reflected in Table 2.

**TABLE 2:** RT-qPCR primer sequences.

Gene name	Forward (5′–3′)	Reverse (5′–3′)
Occludin	CCC​AGG​CTT​CTG​GAT​CTA​TGT	TCC​ATC​TTT​CTT​CGG​GTT​TTC​A
ZO-1	GGG​AAA​ACC​CGA​AAC​TGA​TG	GCT​GTA​CTG​TGA​GGG​CAA​CG
JAM	CAA​GGC​AAG​GGT​TCG​GTG​TA	GCT​GTA​CTG​TGA​GGG​CAA​CG
CYP7A1	GGG​ATT​GCT​GTG​GTA​GTG​AGC	GGT​ATG​GAA​TCA​ACC​CGT​TGT​C
CYP8B1	AAC​AGC​TCA​TCG​GCC​TCA​TC	AAG​GCT​GGC​TTC​CTG​AGC​TT
CYP27A1	CCA​AGG​CAA​GGT​GGT​AGA​GA	CTT​CAT​CGC​ACA​AGG​AGA​GC
SR-B1	AAT​GCT​CCT​TTG​GGT​TAG​GG	GCC​CCC​GAT​ACT​CTG​TTT​G
LDL-R	ATG​CTG​GAG​ATA​GAG​TGG​AGT​T	CCGCCAAGATCAAGAAAG
HMGCR	TGCTGGTGCTATCAAAGG	GCAGATGGGATGACTCGA
ABCA1	TGGACATCCTGAAGCCAG	TTCTTCCCACATGCCCT
BSEP	TCT​GAC​TCA​GTG​ATT​CTT​CGC​A	CCC​ATA​AAC​ATC​AGC​CAG​TTG​T
NTCP	CAA​ACC​TCA​GAA​GGA​CCA​AAC​A	GTA​GGA​GGA​TTA​TTC​CCG​TTG​TG
MRP2	GCT​TCC​CAT​GGT​GAT​CTC​TT	ATC​ATC​GCT​TCC​CAG​GTA​CT
MRP4	TTA​GAT​GGG​CCT​CTG​GTT​CT	GCC​CAC​AAT​TCC​AAC​CTT​T
OATP-1	GTC​TTA​CGA​GTG​TGC​TCC​AGA​T	GGA​ATA​CTG​CCT​CTG​AAG​TGG​ATT
FXR	GGC​AGA​ATC​TGG​ATT​TGG​AAT​CG	GCC​CAG​GTT​GGA​ATA​GTA​AGA​CG
SHP	TGG​GTC​CCA​AGG​AGT​ATG​C	GCT​CCA​AGA​CTT​CAC​ACA​GTG
PPAR-α	CCT​GGA​AAG​TCC​CTT​ATC​T	GCC​CTT​ACA​GCC​TTC​ACA​T
SREBP-1c	CAG​CAG​CAG​TGG​TGG​CAG​TG	GGT​TGC​AGG​TCA​GAC​ACA​GGA​AG
LRH-1	GAA​CTG​TCC​AAA​ACC​AAA​AAA​GG	CTT​CCA​GCT​TCA​TCC​CAA​C
FGFR4	CCC​TTG​GAC​TCA​TCC​TCA​GA	GTG​AAG​TCC​CAA​GGC​CTC​TA
NPC1L1	TGT​CCC​CGC​CTA​TAC​AAT​GG	CCT​TGG​TGA​TAG​ACA​GGC​TAC​TG
GAPDH	AGT​TCA​ACG​GCA​CAG​TCA​AGG	GTC​TTC​TGA​GTG​GCA​GTG​ATG​G

### Statistical Analysis

SPSS (Version 25.0) was used for statistical analysis. All experimental data were expressed as mean ± SEM. Comparisons between two groups and more than two groups in the experiment were done by Student’s *t*-test and one-way analysis of variance (ANOVA), respectively. And *p* < 0.05 was considered statistically significant.

## Results

### Quality Evaluation of Ethanol Extract of RPMP

**FIGURE 1:**
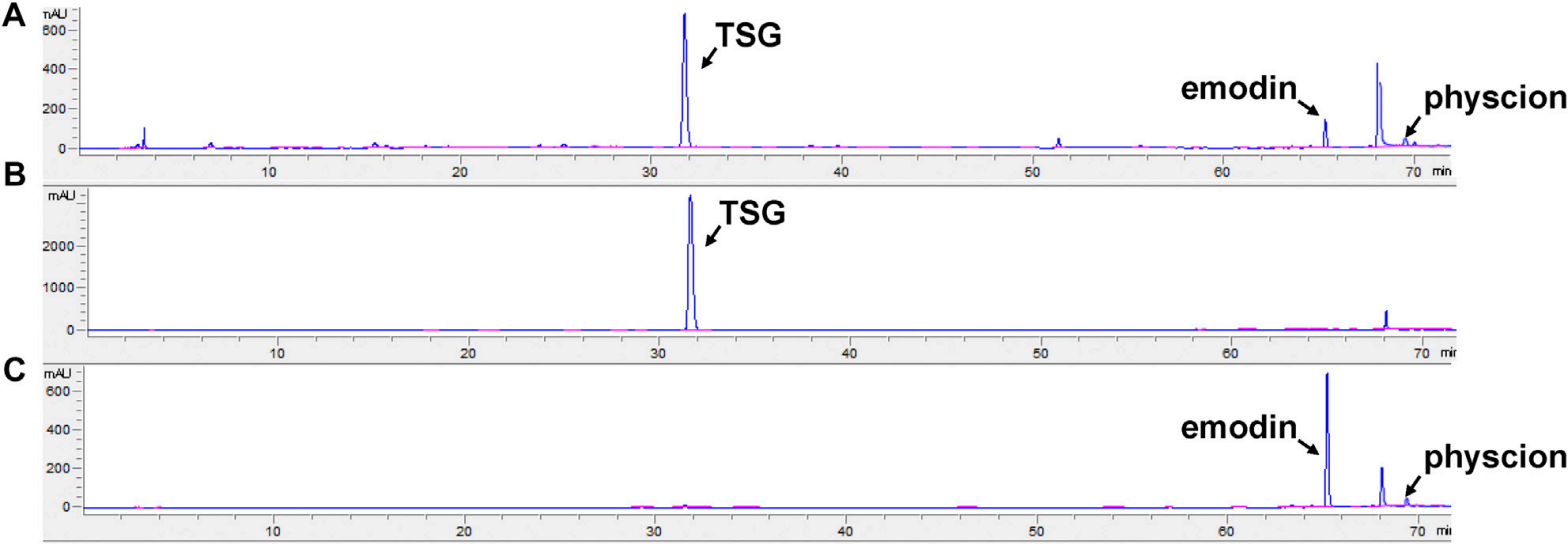
HPLC chromatogram. **(A)** RPMP ethanol extract. **(B)** TSG. **(C)** emodin and physcion.

According to Chinese Pharmacopoeia, the contents of TSG and free anthraquinone (calculated by the total amount of emodin and physcion) in the ethanol extract of RPMP were measured to determine whether they meet the quality standards (Commission, 2020). The quantitative results showed that the contents of TSG and free anthraquinone in the ethanol extract of RPMP were 4.01 and 0.21%, respectively, (Figure 1).

### Dose Selection of RPMP Ethanol Extract

**FIGURE 2:**
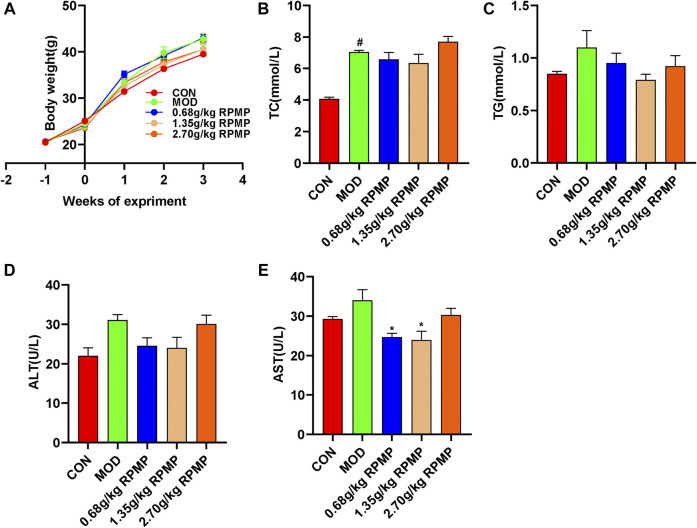
Dose assessment of RPMP ethanol extract. **(A)** Trends in body weight of mice. **(B–E)** Serum biochemical indices of mice. The data were presented as the means ± SEM, ^#^ *p* < 0.05, compared with the CON group; *p < 0.05, compared with the MOD group (*n* = 6).

Prophylactic administration was taken to investigate the effective therapeutic dose of RPMP ethanol extract on serum biochemical parameters in mice under HFD administration. During the 3-weeks administration, there were no significant differences were observed between the dose groups in terms of the trend of body weight changes in the mice (Figure 2A). However, in terms of serum biochemical indicators, although each dose group did not show significant modulating effects compared with HFD mice, the results of each biochemical indexes showed that the 2.70 g/kg dose group was inferior to the 1.35 g/kg dose group in regulating serum lipid metabolism and liver function indexes induced by HFD (Figures 2B–E). The reason why RPMP ethanol extract did not show dose-dependence may be due to the fact that the dose of RPMP exceeded the threshold of treatment, and which disturbed the normal metabolic function of the liver, thus causing abnormal liver biochemical indexes. And during a 42-days study on the lipid regulation of RPMP in SD rats induced by high-fat diet (HFD), compared with middle dose (1.620 g/kg), low dose (0.810 g/kg), and high dose (3.240 g/kg) RPMP showed better effects in regulating liver lipid metabolism (TC, TG, LDL-C, HDL-C); However, in serum biochemical parameters, the medium dose of RPMP showed better results compared to the low and high doses (Li et al., 2012). And the middle dose (140 mg/kg) of liver TC was better than the low dose (70 mg/kg) and the high dose (280 mg/kg) of liver TC indices in 8 weeks Wistar rats treated with RPMP(Lin et al., 2020). And studies have shown that this phenomenon can return to normal after discontinuation of the drug. Therefore, a dose of 1.35 g/kg was chosen as the maximum dose of RPMP ethanol extract intervention in the follow-up experiments.

### RPMP Ethanol Extract Improves Serum Lipid Metabolism

**FIGURE 3:**
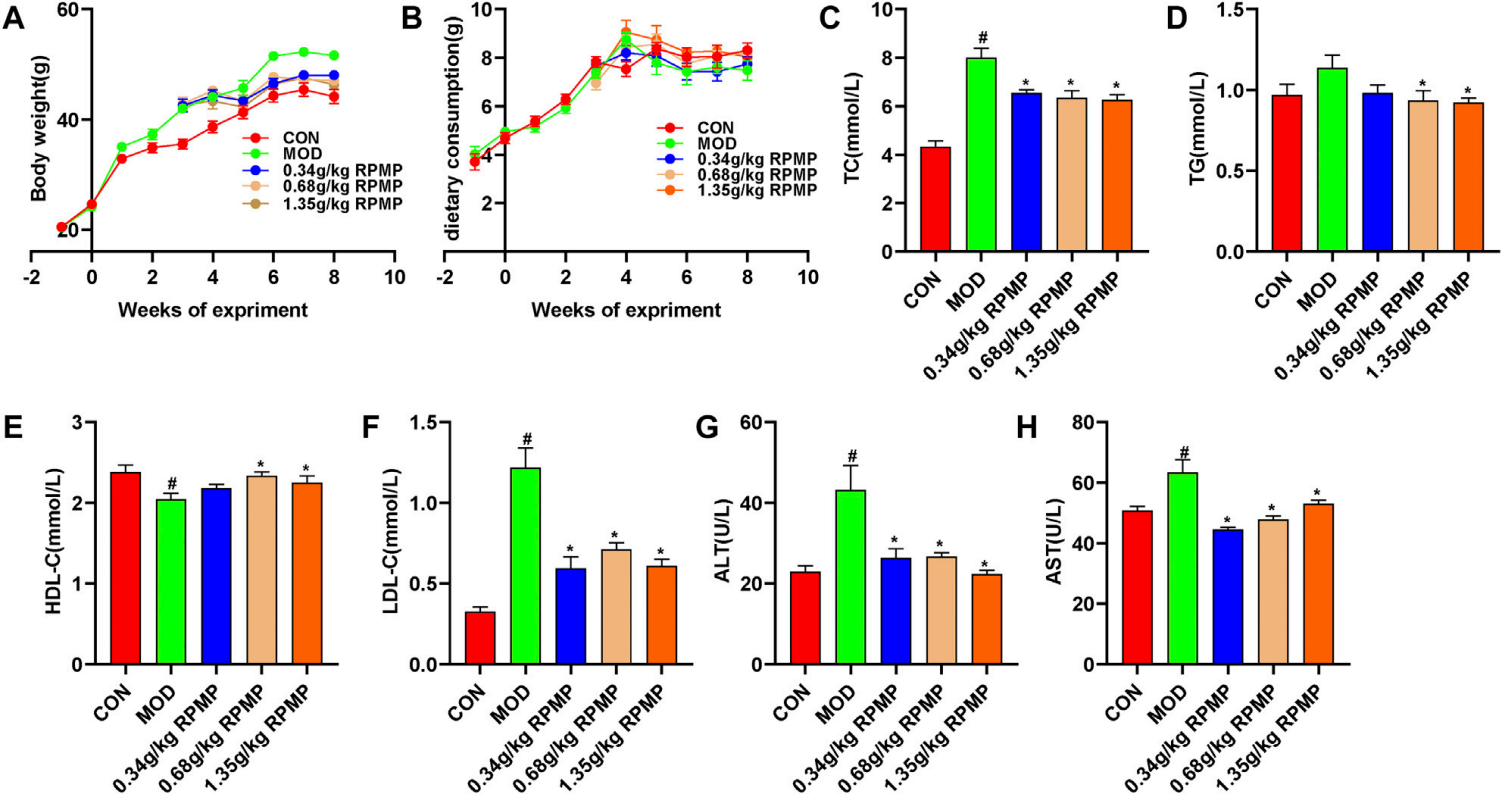
Effects of RPMP ethanol extract on body weight and serum biochemical parameters in HFD-induced mice. **(A)** Trends in body weight of mice. **(B)** Curve of weekly food intake in mice. **(C–H)**, Serum biochemical indices of mice. The data were presented as the means ± SEM, ^#^ *p* < 0.05, compared with the CON group; *p < 0.05, compared with the MOD group (*n* = 8).

Obesity is closely related to the occurrence and development of NAFLD. Mice induced by HFD gained 113.93% body weight at the end of the experiment compared to the beginning, and this trend was significantly suppressed by the intervention of RPMP ethanol extract (Figure 3A). However, there was no significant difference in the food intake of mice between the groups (Figure 3B). HFD induced not only an increase in body weight, but also a significant increase in serum TC, TG, and LDL-C levels. However, significantly decreased HDL-C levels in lipid metabolism in mice. When the mice were treated with RPMP ethanol extract, the increase of serum TC, TG, and LDL-C levels was inhibited, while HDL-C levels were restored. In addition, serum levels of ALT and AST, markers of liver injury, which were significantly elevated in HFD administered mice, were also significantly improved by the intervention of RPMP ethanol extract. And this temporarily provided the ability of RPMP to improve the abnormalities of serum biochemical parameters caused by HFD (Figures 3C–H).

### Effect of RPMP Ethanol Extract on Histopathological Changes of Liver Induced by HFD

**FIGURE 4:**
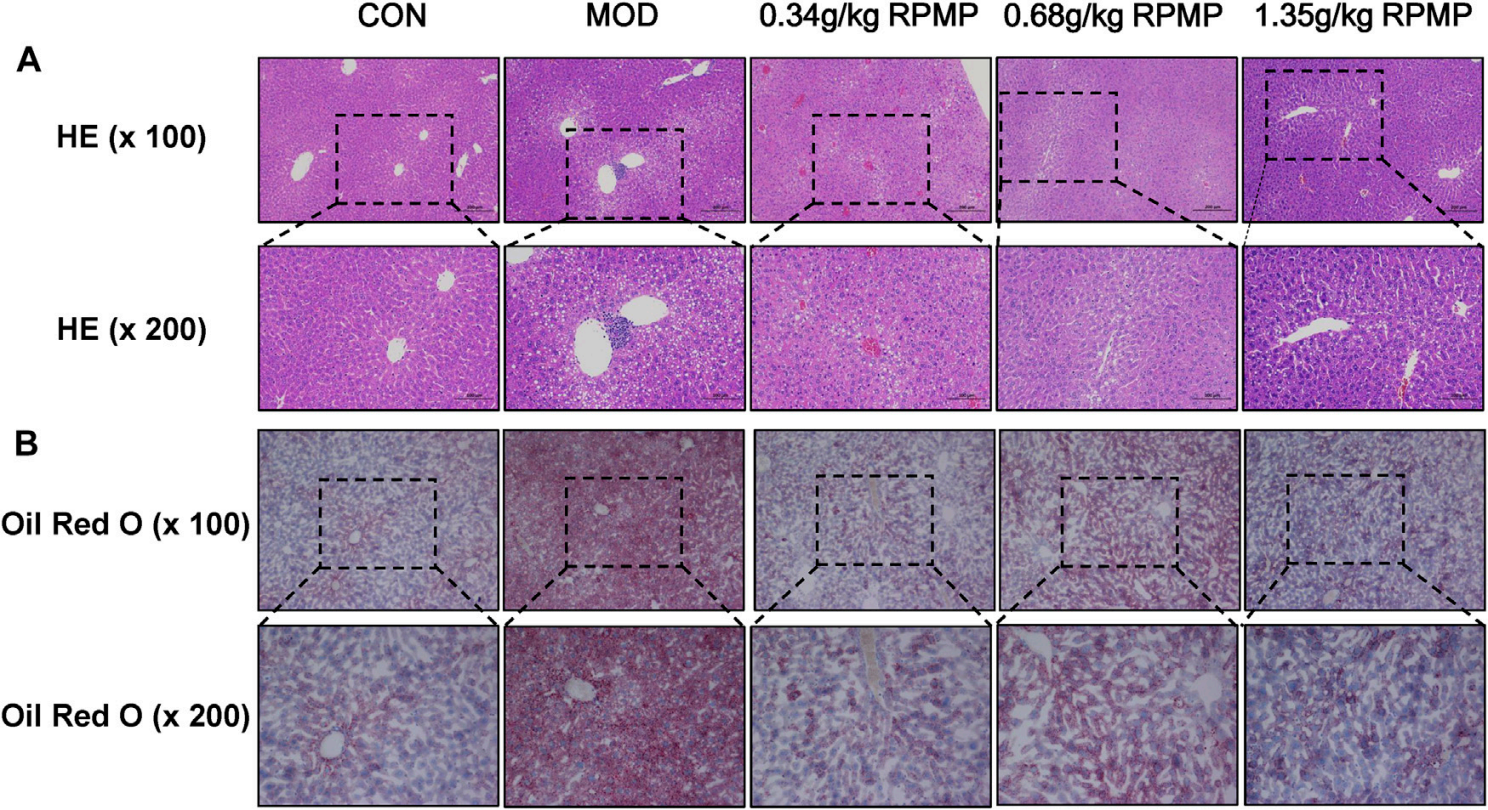
Pathological staining of mouse liver. **(A)** HE staining of liver (×100 and ×200). **(B)** Oil Red O staining of liver (×100 and ×200).

Pathological changes in the liver can directly reflect intrahepatic lipid droplet aggregation and inflammatory infiltration. HE staining observed that ND-fed mice had intact liver lobules, clear hepatic cord structure, rich hepatocyte cytoplasm, normal morphology and structure, no obvious dilatation or extrusion of liver sinusoids, and no obvious inflammation. In contrast, in HFD-fed mice, the tissues exhibited a large amount of hepatocyte steatosis, with round vacuoles of varying sizes visible in the cytoplasm, and focal infiltration of a small number of lymphocytes was seen around the local central vein. With the administration of different doses of RPMP ethanol extract, the hepatocellular steatosis present in the tissues was gradually suppressed, with clear hepatic cord structure, no significant dilatation or extrusion of the hepatic sinusoids, and no significant inflammation (Figure 4A).

Additionally, lipid deposition in the liver was assessed by oil red O staining. The results showed that, compared with ND, a large area of lipid tissue was stained red in the HFD-fed mice, demonstrating the presence of a large amount of lipid accumulation. In contrast, the degree of redness in the tissues decreased in a dose-dependent manner after the administration of RPMP ethanol extract. And these results showed that RPMP could reduce liver lipid deposition to a certain extent (Figure 4B).

### RPMP Ethanol Extract Reduced the Weight of Visceral and Adipose Tissue in HFD-Induced Mice

**FIGURE 5:**
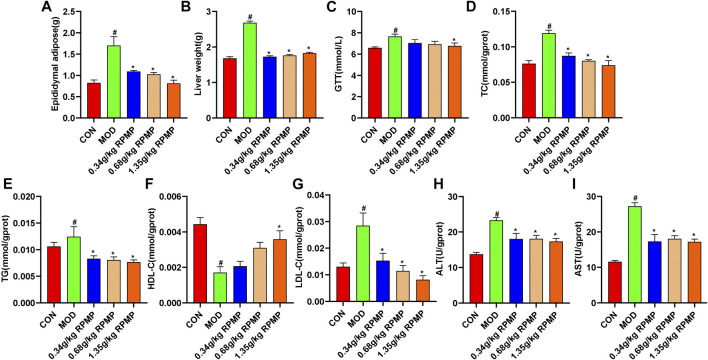
Physiological and biochemical indices of mouse liver. **(A,B)** Liver physiological indicators. **(C)** Fasting blood glucose levels in mice. **(D–I)** Liver biochemical indicators. The data were presented as the means ± SEM, ^#^ *p* < 0.05, compared with the CON group; *p < 0.05, compared with the MOD group (*n* = 8).

The liver and epididymal fat weights of mice were examined, and it was found that the liver and epididymal fat weights of HFD-induced mice increased by 59.51 and 107.15%, respectively, compared with those of ND-fed mice. In contrast, the liver and epididymal fat weight of mice treated with RPMP ethanol extract showed a significant reduction relative to that of mice administered with HFD (Figures 5A,B). Meanwhile, an 8-weeks HFD administration resulted in a 16.54% increase in fasting blood glucose levels (GTT) in mice. In contrast, after RPMP ethanol extract treatment, fasting blood glucose levels in mice decreased, with a 13.31% decrease in the 1.35 g/kg RPMP-dose group (Figure 5C).

### RPMP Ethanol Extract Improves Liver Lipid Metabolism

The liver is the main metabolic organ responsible for lipid and lipoprotein. Compared with ND-fed mice, we found that liver levels of TC, TG, and LDL-C showed an increase in the same way as in serum, while HDL-C also showed a tendency to decrease under the effect of HFD. Undoubtedly, these trends seem to recover in a dose-dependent manner after the intervention of RPMP ethanol extract (Figures 5D–G). Similarly, in HFD-fed mice, the levels of liver function indicators (ALT and AST) were significantly elevated, and which was significantly inhibited by the ethanol extract of RPMP (Figures 5H,I). Accordingly, it was concluded that RPMP ethanol extract could improve the abnormal lipid metabolism parameters and elevated liver function indexes caused by HFD.

### Modulation of Intestinal Microbial by RPMP Ethanol Extract

**FIGURE 6:**
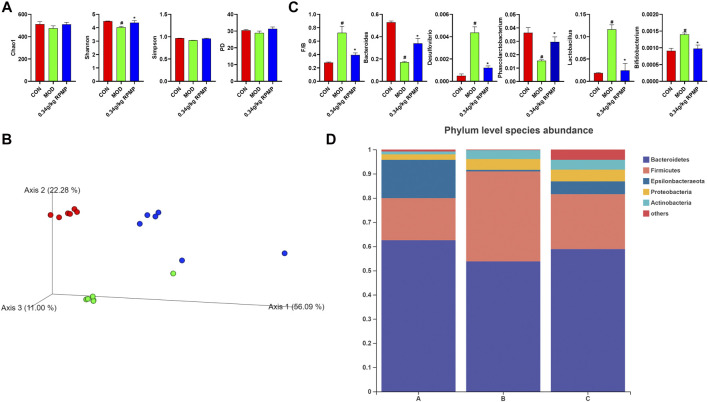
Effect of RPMP on the IM composition of mice. **(A)** α-Diversity Index. **(B)** Principal Co-ordinates Analysis (PCoA). **(C)** Species Abundance. **(D)** Distribution of species at the phylum level. The data were presented as the means ± SEM, #p < 0.05, compared with the CON group; *p < 0.05, compared with the MOD group. Group A: CON group. Group B: MOD group. Group C: 0.34 g/kg RPMP ethanol extract group. (n = 6).

The unique anatomical position of the intestine in relation to the liver makes the liver the first organ exposed to intestinal-derived factors, and thus the composition of the microbiota was examined next. A healthy microbial community was characterized by rich microbial diversity, which can reflect the stability and reduction ability of the ecosystem. The Chao 1 and Shannon indices, which were used to reflect microbial community diversity, showed a tendency to decrease under the effect of HFD compared to ND-fed mice, where a 10% loss of Shannon index was observed, which was suppressed when RPMP ethanol extract was given (Figure 6A). Meanwhile, Principal Co-ordinates Analysis (PCoA) results based on weighted Unifrac distances showed that mouse samples administrated with HFD were divided into different clusters with ND mice, and the administration of RPMP ethanol extract appeared to be reshaping the intestinal microbial community disrupted by HFD (Figure 6B).

**FIGURE 7:**
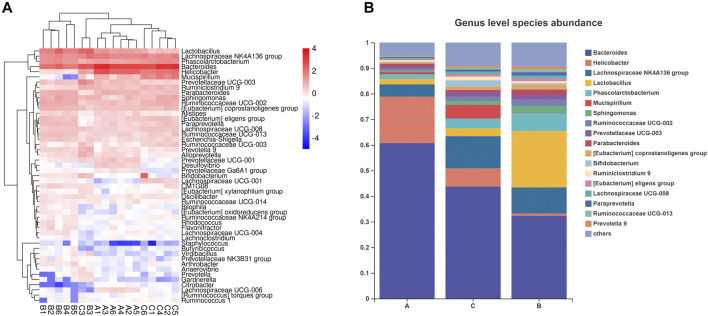
Effect of RPMP on the IM composition of mice. **(A,B)** Distribution of species at the genus level. Group A: CON group. Group B: MOD group. Group C: 0.34 g/kg RPMP ethanol extract group. (*n* = 6).

*Bacteroides* abundance was reduced and *Firmicutes* abundance was increased in the microbial community of mice subjected to HFD. In contrast, the increased *Firmicutes/Bacteroides* (F/B) ratio was reduced under RPMP ethanol extract intervention (Figures 6C,D). The genus-level clustering heat map showed that separate clusters were formed between each group, but the samples from the RPMP ethanol extract were not completely separated from the ND-fed mice compared to the HFD-fed mice (Figure 7A). Further analysis was conducted and found that *Bacteroides* and short-chain fatty acids (SCFAs) producing bacteria *Phascolarctobacterium* exhibited a reduction in HFD intervention, where butyrate in SCFAs was able to reduce fat deposition and insulin resistance through lipoprotein-activated β-oxidation and cholesterol transport (Elvira-Torales et al., 2019). However, the reduced abundance of *Bacteroides*, *Phascolarctobacterium* were recovered after the administration of RPMP ethanol extract (Figures 6C, 7B). *Desulfovibrio* is a gram-negative bacterium that produces endotoxins and has been shown to increase intestinal permeability and production of enterogenic factors (mainly LPS) (Zhao et al., 2019). The increase in *Desulfovibrio* abundance caused by HFD was effectively reduced after RPMP ethanol extract treatment (Figure 6C).

**FIGURE 8:**
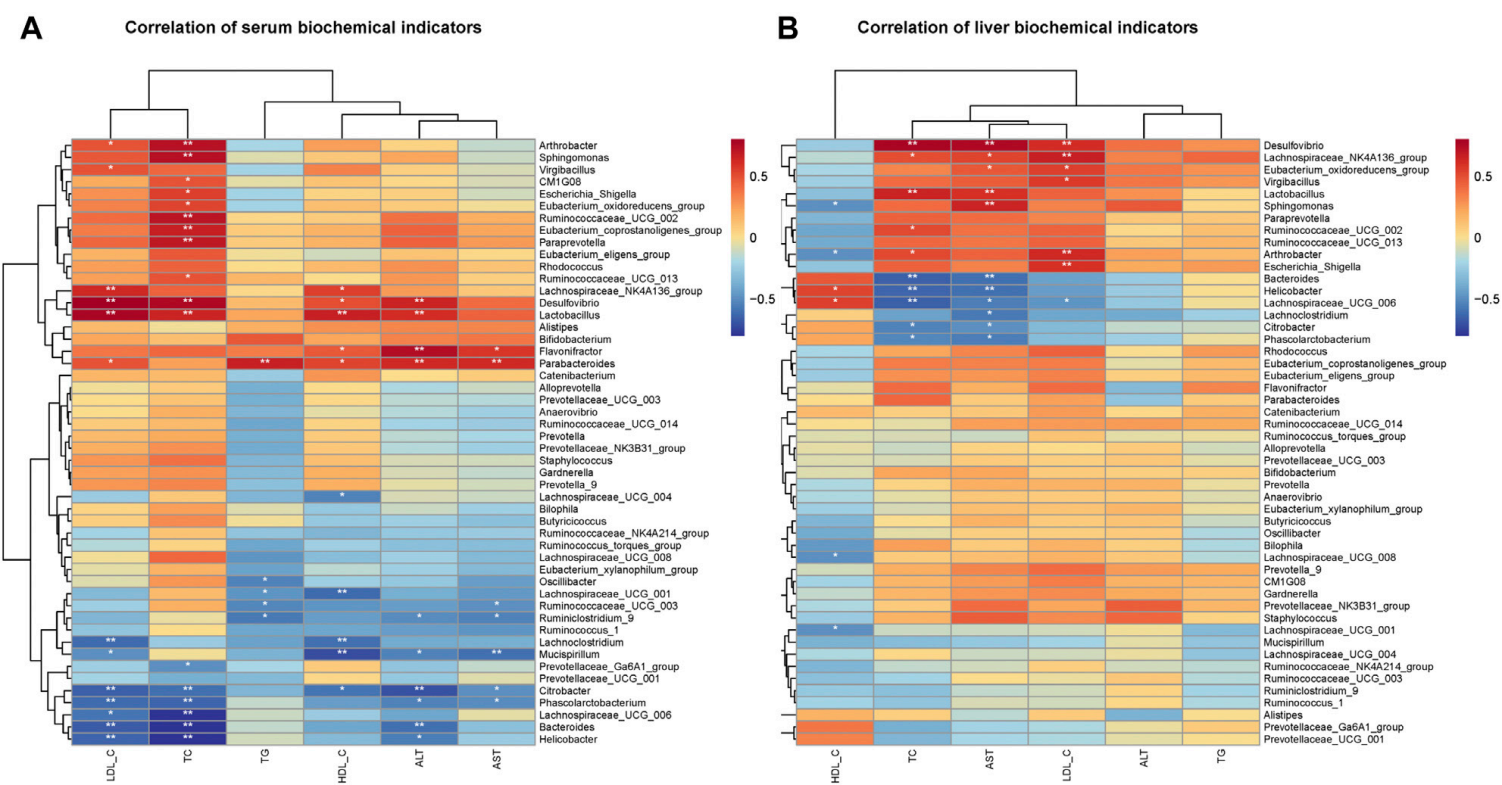
Correlation analysis of IM with serum and liver biochemical parameters in mice. **(A)** Correlation analysis of serum biochemical indicators. **(B)** Correlation analysis of liver biochemical indicators.

Since the intervention of RPMP ethanol extract can affect the biochemical parameters and the structural composition of IM in mice administered with HFD, Pearson’s correlation was used to find the relationship between biochemical indicators and IM species in mice. As observed for changes in the abundance of IM species, there was a negative correlation between the decrease in the abundance of *Bacteroides* and *Phascolarctobacterium* and the changes in serum and liver biochemical parameters caused by HFD (Figures 8A,B). Moreover, in this process, *Ruminococcaceae UCG-002, Ruminococcaceae UCG-013, Ruminiclostridium 9, Ruminococcaceae UCG-003, Ruminococcaceae UCG-014*, and other Species of the *Ruminococcaceae* show inconsistent relationships, which was thought to be due to the fact that the *Ruminococcaceae* was a very heterogeneous family -containing both harmful and beneficial bacteria (Kong et al., 2019). Therefore, it was concluded that RPMP ethanol extract has a remodeling effect on the disorder of IM structure caused by HFD.

### Regulation of Intestinal Pathology and Intestinal Epithelial Barrier-Related Genes by RPMP Ethanol Extract

**FIGURE 9:**
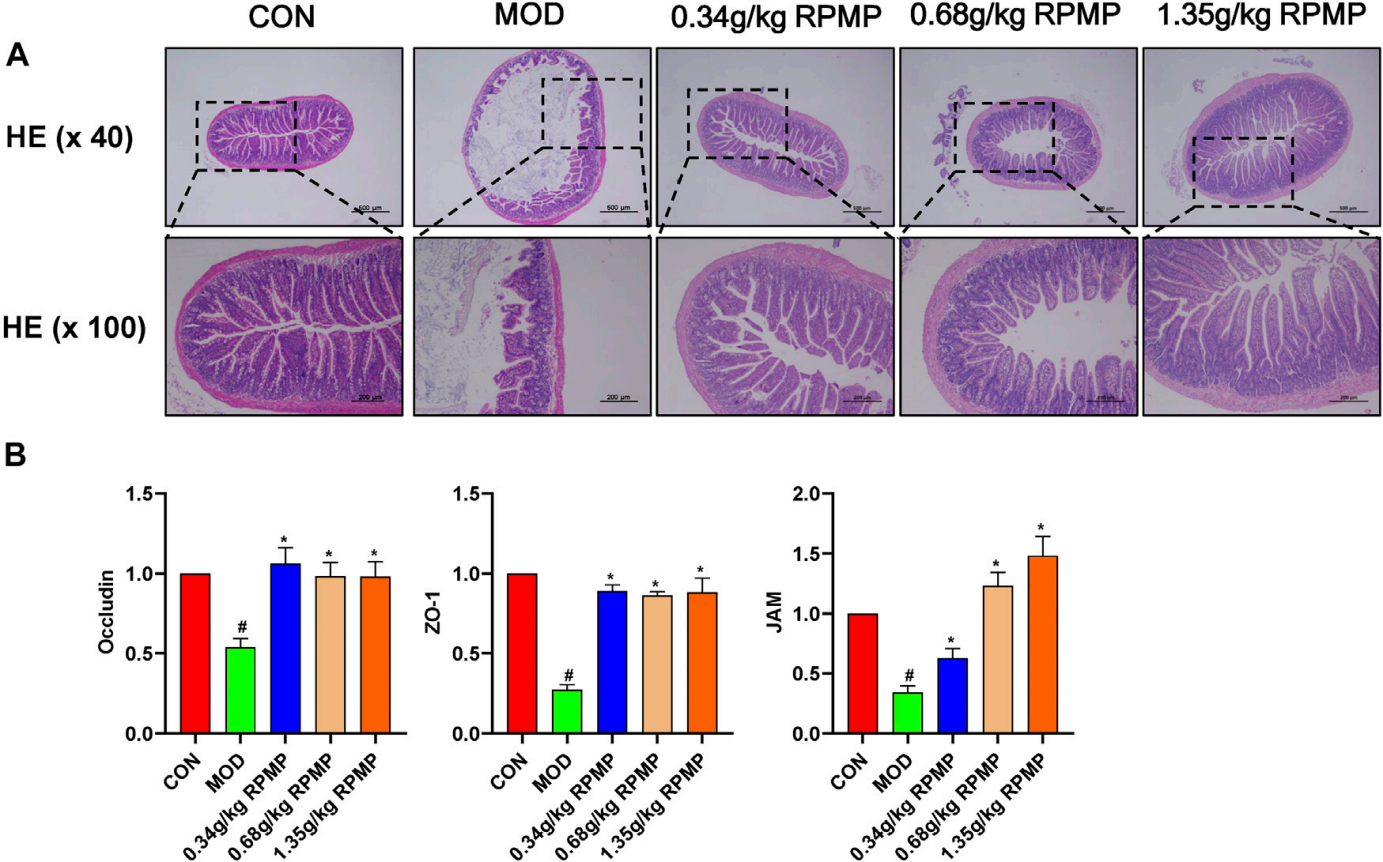
Intestinal epithelial barrier. **(A)** Colonic pathology in mice (×40 and ×100). **(B)** Effect of RPMP ethanol extract on intestinal barrier-related genes. The data were presented as the means ± SEM, ^#^ *p* < 0.05, compared with the ND group; *p < 0.05, compared with the HFD group (*n* = 6).

A major consequence of HFD induction was an increase in intestinal permeability. Disruption of the intestinal epithelial barrier increases the risk of liver exposure to enteric-derived factors. HE staining of the intestine showed that the intestinal wall of mice under the effect of HFD was obviously damaged, and the villi were shed and highly reduced. The morphological structure of the villi was restored to varying degrees after the administration of RPMP ethanol extract (Figure 9A).

The mRNA expression levels of tight junction (TJ) molecules, such as zonula occluden-1 (ZO-1), Junction adhesion molecule (JAM), and Occludin, which serve as markers of intestinal epithelial barrier integrity, were significantly downregulated under the management of HFD. And, the reduced gene expression levels were in line with the changes in IM structure. And as expected, administration of RPMP ethanol extracts prevented the reduction of mRNA expression levels of ZO-1, JAM, and Occludin (Figure 9B).

### Effect of RPMP Ethanol Extract on Bile Acid Metabolism

The ability of adjacent intestinal epithelial cells to form TJ was crucial for the formation and maintenance of the intestinal epithelial barrier. As a dynamic regulatory structure, it plays a double-sided role, preventing potentially harmful intestinal-derived factors from entering the body’s circulation, while at the same time providing the body with the absorption of essential nutrients and ions, etc. However, several results have shown that the function of TJ was influenced by the level of BAs in the organism (Münch et al., 2007; Raimondi et al., 2008). Increased levels of ZO-1, Occludin protein expression were found in the intestine of HFD-fed *F11r* ^*−/−*^ mice after sequestration of BAs (Gupta et al., 2020). And, in studies of animal models of NAFLD, supplementation with BAs and treatment of BAs-related receptors have also been used (McMahan et al., 2013; Wang et al., 2018). Thus, the composition structure of BAs in mouse feces was next examined.

The HFD-induced pool of mouse BAs was more significantly altered in terms of CA, CDCA, TCA, TCDCA, TLCA, GCA, GDCA, T-α-MCA, T-β-MCA, and other species. Among them, an increase in CA concentration could lead to an increase in the abundance of *Firmicutes* and a decrease in the abundance of *Bacteroidetes* (Staley et al., 2017). Also in patients with cirrhosis, an increase in the potentially pathogenic bacteria *Enterobacteriaceae* showed a positive correlation with the concentration of CDCA present (Kakiyama et al., 2013). Not only that, the study showed that the IM structure of liver tumor mice was significantly altered, in which DCA, TCA, TCDCA, and TLCA together contributed to the development of liver tumors. And this confirms the mutual crosstalk between BAs and IM.

**FIGURE 10:**
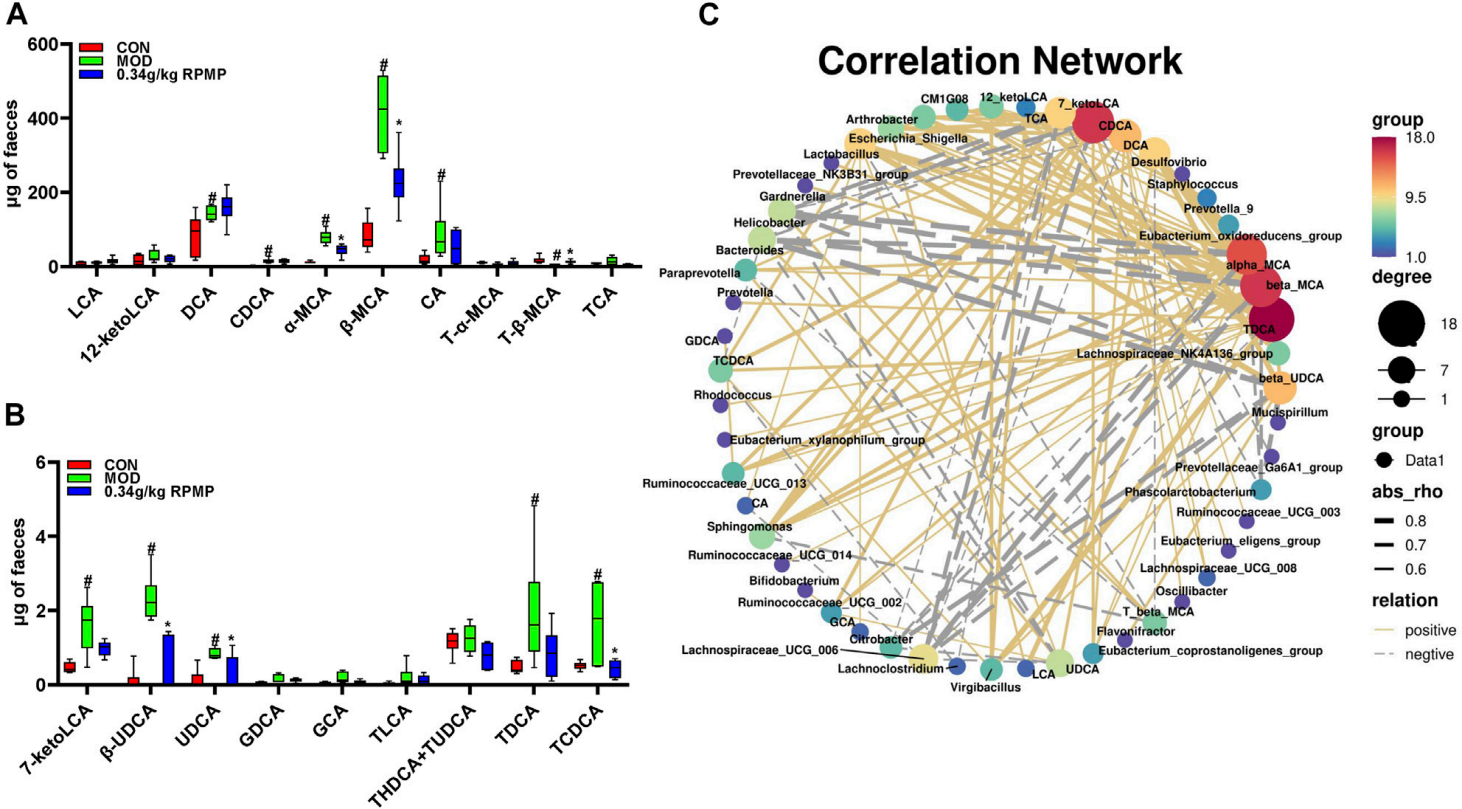
Structural components of BAs pool. **(A,B)** Effect of RPMP ethanol extract on BAs species. **(C)** Correlation network diagram of IM species with BAs. The data were presented as the means ± SEM, ^#^ *p* < 0.05, compared with the CON group; *p < 0.05, compared with the MOD group (*n* = 6).

T-β-MCA downregulated fibroblast growth factor 15 (FGF15) expression through inhibition of intestinal farnesoid X receptor (FXR) and, induced fibroblast growth factor receptor 4 (FGFR4)-dependent cytochrome P450 family seven subfamily A member 1 (CYP7A1) activation through inhibition of JUN transcriptional activity, thereby regulating the increased biosynthesis of BAs. In contrast, in mice, low levels of T-β-MCA activated intestinal FXR signaling, which explained the abnormally elevated intestinal FXR expression in our results (Sayin et al., 2013). Meanwhile, T-α-MCA and T-β-MCA were formed as α-MCA and β-MCA by the deconjugation of bile salt hydrolases (BSH). *Bacteroides, Clostridium, Firmicutes, Bifidobacterium, Lactobacillus, Listeria*, and many other microorganisms are able to activate the production of BSH. Thus, it was suggested that the abnormal elevation of α-MCA and β-MCA expression in HFD-induced mice might be due to the disordered IM structure (Figure 10A). And this may be responsible for the low level of T-β-MCA as well as the abnormal expression of intestinal FXR genes. These BAs species, which were altered by HFD administration, have recovered after treatment with RPMP ethanol extract (Figures 10A,B).

**FIGURE 11:**
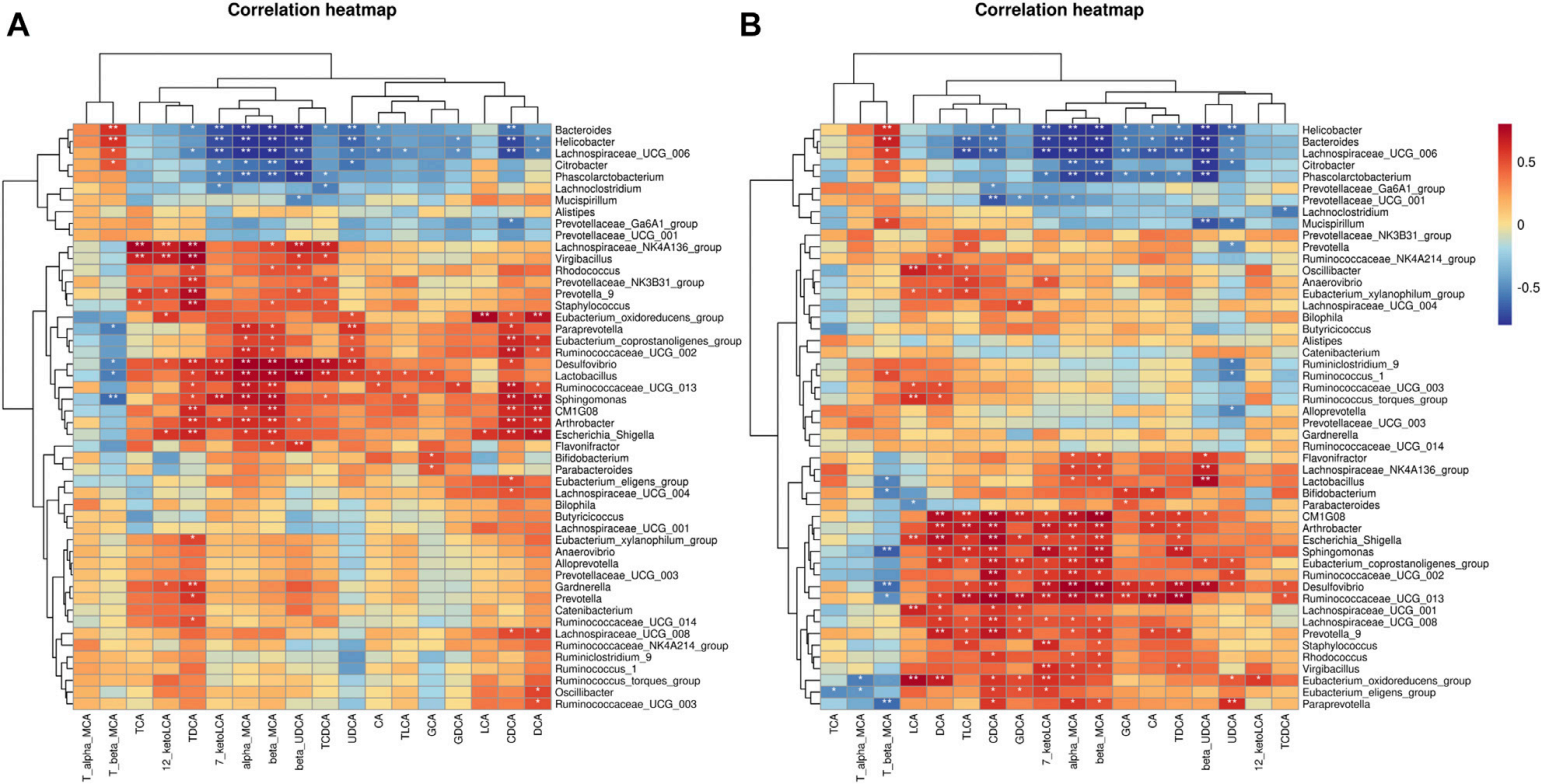
Correlation analysis of BAs species with IM species in mice. **(A)** Based on Pearson's correlation coefficient. **(B)** Based on Spearman's rank correlation coefficient.

Since the administration of HFD caused a change in the composition of the IM and BAs pools in mice, correlation analysis was used to examine whether there was a dynamic relationship between the altered IM and BAs. We noticed a positive correlation between hydrophobic BAs –LCA, DCA, CDCA, CA, and UDCA, which are cytotoxic, and the abundance of most IM species (Figures 11A,B). Further, TDCA, alpha-MCA, beta-MCA, and CDCA were found to have more nodes in the correlation network compared to other species (Figure 10C). TDCA is a conjugate of deoxycholic acid and taurine in the form of sodium salt. In NASH patients, a more than 5-fold increase in TDCA metabolite levels compared to normal organism levels was observed (Lake et al., 2013). Meanwhile, abnormally elevated serum TDCA levels were found in high-fat diet-induced NASH mice (He et al., 2021). In our results, we also observed a significant increase in TDCA levels in mice induced by a high-fat diet. In addition, the cytotoxic hydrophobic bile acid CDCA also has multiple nodes (Figure 10C). A recent study also showed that increased transmission of CDCA to the colon, induced by a high-fat diet, disrupts the intestinal epithelial barrier (Gupta et al., 2020). We also observed a significant increase in the level of CDCA in the feces of mice induced by a high-fat diet, and the potency of FXR activation by endogenous bile acids is CDCA > LCA = DCA (Gonzalez et al., 2016). And this may explain the abnormal elevation of intestinal FXR gene expression in mice induced by a high-fat diet.

### Effect of RPMP Ethanol Extract on Bile Acids and Genes Related to Bile Acid Metabolism

**FIGURE 12:**
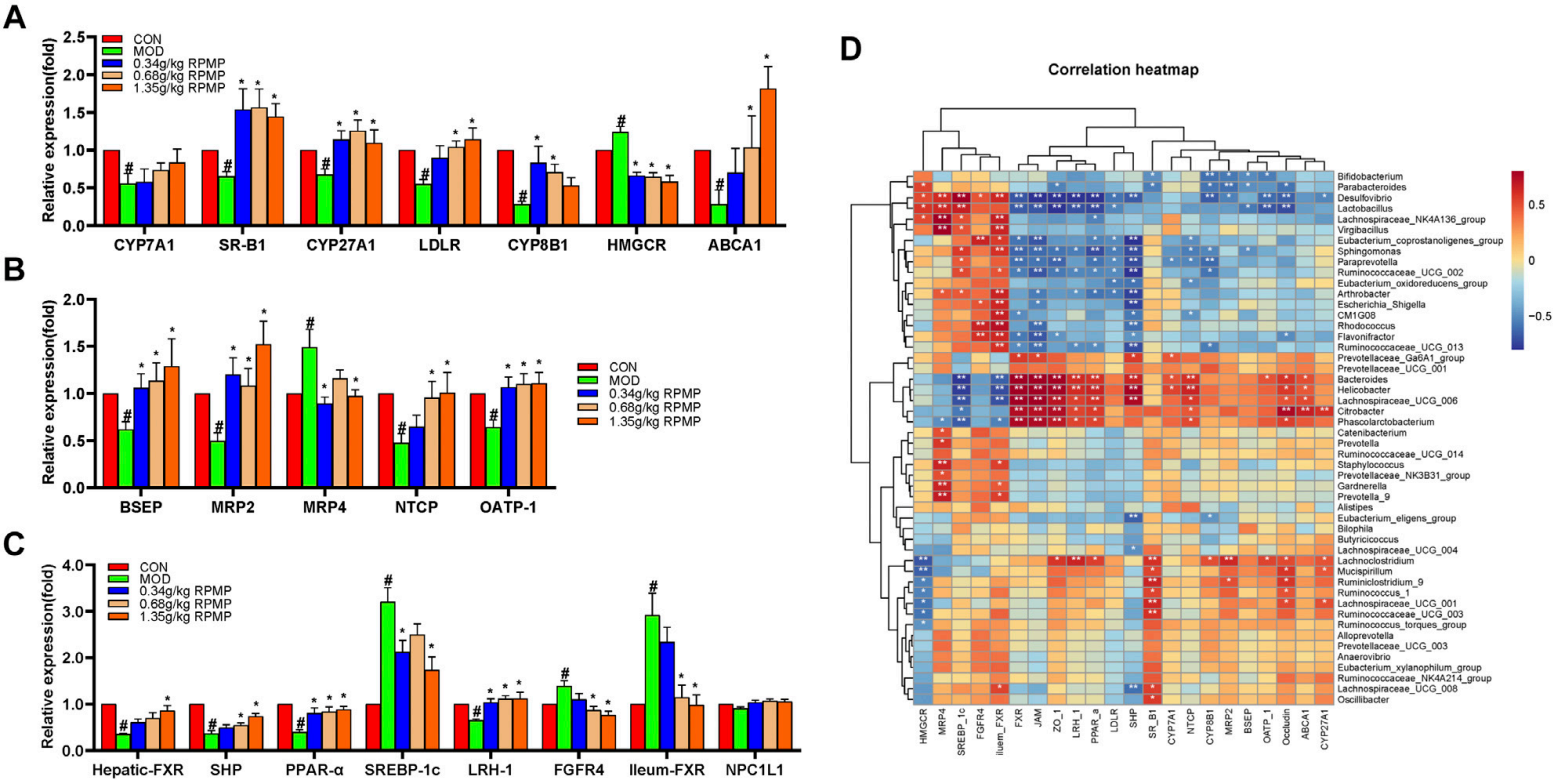
Effect of RPMP ethanol extract on genes related to BAs metabolism. **(A)** Genes related to BAs synthesis. **(B)** Genes related to BAs transport. **(C)** BAs-regulated related genes. **(D)** Correlation analysis of IM species with BAs metabolism and intestinal barrier-related genes in mice. The data were presented as the means ± SEM, ^#^ *p* < 0.05, compared with the CON group; *p < 0.05, compared with the MOD group (*n* = 8).

Cholesterol is a substrate for the synthesis of BAs, and the mRNA expression levels of ATP binding cassette subfamily A member 1 (ABCA1), scavenger receptor class B member 1 (SR-B1), and low-density lipoprotein receptor (LDLR), key genes responsible for transporting excess cholesterol from the diet and surrounding tissues to the liver, were significantly reduced by the intervention of HFD, while the expression of 3-hydroxy-3-methylglutaryl-CoA reductase (HMGCR), responsible for the synthesis of cholesterol in the liver, was significantly increased. And, the mRNA expression of CYP7A1, the rate-limiting enzyme in the classical pathway of BAs synthesis, and mitochondrial sterol 27-hydroxylase (CYP27A1), which initiates the first step of the alternative synthesis pathway, was indeed significantly reduced (Figure 12A). That can be an important cause of cholesterol buildup in the liver.

In addition, bile salt export pump (BSEP), multidrug resistance protein 2 (MRP2), sodium taurocholate cotransporting polypeptide (NTCP), and organic anion transporting polypeptide (OATP-1) were significantly reduced in mice treated with HFD, except for the increased expression of the multidrug resistance protein 4 (MPR4) gene (Figure 12B). These genes were involved in the transport, release, and reabsorption of BAs *in vivo*.

The synthesis of BAs is regulated by a negative feedback mechanism. Furthermore, the expression of FXR, small heterodimer partner (SHP), liver receptor homolog 1 (LRH-1), and FGFR4 was significantly decreased, while sterol regulatory element-binding protein 1c (SREBP-1c) was significantly increased under the effect of HFD. On the other hand, the expression of FXR in the intestine did show an increasing trend (Figure 12C).

In addition, Niemann-Pick C1-Like 1 (NPC1L1) plays an important role in intestinal cholesterol absorption. However, in our results, the mRNA expression levels of NPC1L1 did not differ significantly between groups and even showed a slight tendency to decrease in the HFD-induced mice (Figure 12C). And the content of NCP1L1 protein in the intestinal epithelium of mice, also induced by a high-fat diet, was significantly reduced compared with that of a low-fat diet (Losacco et al., 2018). In a study examining the effects of a high cholesterol diet on the expression of NCP1L1 and intestinal transporters in rats and mice, the level of NCP1L1 mRNA in the ileum tissue of the mice was significantly reduced compared with that of the control group (Kawase et al., 2016). And in patients with hyperlipidemia treated with cholesterol synthesis by HMGCR inhibitor Atorvastatin, the level of NPC1L1 gene expression was significantly increased. At the same time, mice fed a cholesterol-free high-fat diet were found to inhibit both the cholesterol efflux transporter and the cholesterol absorption transporter NPC1L1, as well as the ratio of cholesterol absorption (Tremblay et al., 2011). Therefore, it was speculated that the decrease of NPC1L1 after ingestion of HFD may be due to the response mechanism of the body to supersaturated cholesterol. And decreased NPC1L1 disrupts the hepatic-intestinal circulation of cholesterol, which may account for the HFD-induced increase in the expression of hepatic cholesterol synthesis gene-HMGCR in mice.

The correlation results showed that there was an association between these changed gene expression levels and IM species. *Bacteroides and Phascolarctobacterium* were positively correlated with the expression of hepatic-FXR, JAM, ZO-1, LRH-1, peroxisome proliferator-activated receptor alpha (PPAR-α), NTCP, and Occludin genes which were statistically significant; and negatively correlated with ileum-FXR, SREBP-1c which were statistically significant (Figure 12D).

In contrast, after supplementation with RPMP ethanol extract, the tendency of genes related to BAs synthesis, transport, and regulatory functions was differentially suppressed in HFD-managed mice.

## Discussion

Obesity is considered to be an independent risk factor for NAFLD and it was significantly associated with metabolic disorders (Li et al., 2016). In the study of diet-induced obesity and related symptoms (hyperglycemia, hyperlipidemia, etc.), four strains of mice -named km, ICR, C57BL/6, and BALB/c were usually used as model animals. A study inspecting the performance of these four mice under HFD administration showed that km mice did better than the other three mice in terms of the changes in body weight and liver function indicators (Li et al., 2020). Considering that body weight can be used as an external phenotypic indicator of obesity, km mice were chosen as model animals in this study. And the weight of the mice managed by the ND pattern was 0.85 times that of the HFD-induced mice which had increased 79.45% significantly at the end of this experiment.

The “Chinese Pharmacopoeia” records that *Polygoni Multiflori Radix Praeparata*, with the efficacy including but not limited to melting turbidity and reducing lipid, can be used in the treatment of hyperlipidemia clinically (Commission, 2020). Epidemiological and clinical studies have shown that hyperlipidemia caused by lipid metabolism disorders was often accompanied by the occurrence and development of NAFLD (Zhou et al., 2019). While serum biochemical indicators can reflect the body’s metabolic status directly. At the end of the experiment, serum TC and TG levels of the HFD-induced mice increased by 85.02 and 17.5%, respectively, this upward trend of which had been curbed after the intervention of ethanol extract of RPMP. High-density lipoprotein (HDL) participates in the reverse transport of cholesterol in the organism from lipid-laden macrophages in peripheral tissues to the liver (Robinson, 2010). This process completes the catabolism of cholesterol. HDL can also inhibit the inflammatory reactions caused by LDL-derived oxidized lipids. HDL-C in a low level was deemed to be a factor that can increase the risk of metabolic syndrome (Santos et al., 2009). However, under the intervention of HFD, the serum HDL-C level of mice decreased by 14.06%, and the ethanol extract of RPMP could make it return to normal levels. Furthermore, a cross-sectional study indicated that the increase of LDL-C levels within the normal range was independently associated with the increased risk of NAFLD (Sun et al., 2016). The serum LDL-C level was significantly reduced after the intervention of ethanol extract of RPMP, the decrease of which in the high-dose group was up to 50%, while this level was increased 274.61% in HFD mice. In addition, serum ALT and AST levels are the most sensitive indicators of cell damage for the diagnosis of liver diseases. And with the intervention of RPMP ethanol extract, the elevated serum ALT and AST levels were reduced by an average of 41.71 and 23.49% under HFD feeding.

As an “endocrine organ,” adipose tissue regulates nutritional balance and stores energy through complex interactions with the microenvironment. However, not all adipose tissues have the same characteristics. After the removal of the perinephric and epididymal visceral fat (PEVF) during surgery, the insulin resistance in the liver and peripheral tissues was reversed (Gabriely et al., 2002). In rodent models, PEVF also showed harmful effects on glucose and fat metabolism in the liver (Ben-Shlomo et al., 2012). And the 8-weeks HFD management resulted in a significant increase in the weight of epididymal visceral fat in mice. Meanwhile, the expression levels of PPAR-α and peroxisome proliferator-activated receptor gamma (PPAR-γ) whose function was to regulate fatty acid oxidation, lipid homeostasis, and insulin sensitivity, showed the opposite trend in the epididymal visceral adipose tissue of an HFD-induced steatohepatitis model mouse (Zhou et al., 2017). While visceral obesity leads to a reduction in the glucose uptake pathway regulated through insulin and was associated with insulin resistance (Huang, 2009). The average liver weight of HFD-induced mice was 1.6 times that of ND-fed mice, and the fasting blood glucose levels of HFD-induced mice were significantly increased. The HE staining and oil red O staining of liver tissues also illustrated the severe fatty deformation of the liver. At the same time, the disordered lipid metabolism and liver function indicators indicated that the liver of mice under HFD management was damaged. However, intervention with RPMP ethanol extract was able to ameliorate HFD-induced abnormalities in lipid metabolism and liver function impairment.

As an important factor in regulating the structure of IM –diet is closely related to our lives. The PCoA results in HFD-managed mice and ND-fed mice formed different clusters, which also confirms the opinion above. Moreover, both the decrease in IM diversity and the changes in species abundance demonstrate that the intervention of HFD leads to changes in the ecological structure of the intestinal to some extent. Nevertheless, intestinal dysbiosis was acutely relevant to the occurrence and development of NAFLD. The IM of HFD administered mice was obviously separated from ND-fed mice in species clustering analysis at the genus level, while administration of RPMP ethanol extract resulted in closer IM clustering with ND mice, indicating the ability of RPMP ethanol extract to improve the structure of dysregulated IM. In addition, *Bifidobacterium* and *Lactobacillus*, which were often considered as probiotics, showed an increasing trend under the management of HFD, and a correlation between the abundance of *Lactobacillus* in obese patients was also studied (Armougom et al., 2009). Thus, it was speculated that this may be a compensatory effect of the organism caused by the long-term intake of the HFD diet in mice. Indeed, overgrowth and altered diversity of intestinal microbial populations can lead to intestinal inflammation and disruption of the intestinal barrier (Albillos et al., 2020). Abnormalities in the microbial composition of the colon in mice managed on a high-fat or fiber-deficient diet resulted in increased bacterial penetration and reduced mucosal layer thickness, as well as redistribution of tight junction proteins of the intestinal epithelial barrier (Luck et al., 2015; Desai et al., 2016; Schroeder et al., 2018). Moreover, in C57BL/6J mice fed a high-fat diet, the altered microbiota directly led to disruption of the intestinal epithelium and vascular barrier (Mouries et al., 2019). The mRNA expression levels of ZO-1, JAM, and Occludin were also found to be correlated with the abundance of intestinal microorganisms (Figure 12D).

As a special “metabolic organ,” IM provides essential nutrients to the organism, while metabolites from IM or Enterogenic factors from bacteria themselves can also enter the portal circulation through the intestinal epithelial barrier. In order to deal with these potential inflammatory factors, the liver contains a large number of resident immune cells and other non-parenchymal cells. Excessive activation of such cells can cause severe liver damage. Maintaining the integrity of the intestinal epithelial barrier was one of the effective methods to control the participation of intestinal factors in body circulation (Liu et al., 2020). When intestinal epithelial permeability-deficient mice were given HFD administration, there was more severe steatosis than in ND fed mice; and the subsequent phenomenon –IM structural imbalance in F11r^−/−^ mice and the inflammatory response in their body further confirm the role of the intestinal epithelial barrier in organisms (Rahman et al., 2016). Actually, intestinal epithelial cells are connected to each other by intercellular junctional complexes, including TJ, adherens junctions, bridging granules, and gap junctions (Turner, 2009). These are material and structural bases that constitute the intestinal epithelial barrier with selective permeability. Among them, TJ is a highly dynamic and complex structure composed of more than 50 proteins, which can seal adjacent epithelial cells. And under the stimulation of inflammation and other pathological conditions, TJ shrinks result in the permeability between intestinal epithelial cells increasing, which promotes the translocation of intestinal-derived factors (Cui et al., 2019). And fibrils formed by many transmembrane proteins in TJ pass through the plasma membrane and interact with proteins in neighboring cells. JAM is one of the important single transmembrane proteins, among which JAM-A can regulate the epithelial barrier function. And the content of JAM-A in the colon tissue of NAFLD subjects was lower while the inflammation index was higher (Rahman et al., 2016). The transmembrane proteins JAM-A, Occludin, and claudin proteins can be anchored in the cytoplasm by interacting with the plaque protein ZO-1 (Itoh et al., 1999). Under the intervention of HFD, the mRNA expression levels of JAM, ZO-1, and Occludin in mice were significantly down-regulated. However, when FXR receptor agonists (there are a large number of natural FXR agonists and antagonists *in vivo*) were given to mice with colitis, the intestinal permeability of the mice was improved, and the intestinal epithelial cell pro-inflammatory factors’ expression was suppressed (Gadaleta et al., 2011).

Dysregulation of cholesterol homeostasis has been shown to occur in NAFLD (Ioannou, 2016). In the lipid metabolism pathway, LDLR is a surface receptor present in a variety of tissues and cells, which mediates the binding and endocytosis of apolipoprotein B and E containing lipoproteins, LDL in particular (Xin et al., 2013). HFD was more likely to induce NAFLD in low-density lipoprotein receptor knock-out (LDLR^−/−^) mice, and the level of LDLR in the NASH model was significantly reduced (Wang et al., 2012; Garcia-Jaramillo et al., 2019). And the reason was that the clearance rate of cholesterol in the body was impaired. In contrast, the expression level of LDLR in HFD mice showed a dose-dependent increase after administration of RPMP ethanol extract. Most cells in peripheral organs do not have the ability to completely decompose cholesterol, and often need to enter the liver metabolism through the reverse cholesterol transport process undertaken by HDL (Gottlieb and Canbay, 2019). In this process, ABCA1 transports cholesterol in peripheral tissues to apolipoproteins, and the cell surface HDL receptor SR-B1 promotes cholesterol reverse transport to the liver (Oram and Lawn, 2001). However, the accumulation of liver lipids in NASH patients was related to the decrease of ABCA1 expression level, and up-regulation of SR-B1 expression can promote the reverse transport of liver cholesterol and accelerate cholesterol metabolism (Yang et al., 2010; Xin et al., 2013). In addition, HMGCR, a rate-limiting enzyme in endogenous cholesterol synthesis, has increased expression in NAFLD and NASH, and the degree of increase was related to the level of free cholesterol and the severity of liver disease-related (Min et al., 2012). Thus, reducing the production of endogenous cholesterol and increasing the efficiency of cholesterol reaching the liver from peripheral tissues for metabolism may be the first step for RPMP ethanol extract to improve the abnormal cholesterol metabolism caused by HFD. The biosynthesis of BAs (both classical and alternative pathways) is also one of the main steps for the liver to eliminate cholesterol. Under normal conditions, more than 75% of BAs *in vivo* are produced by classical pathways regulated by various enzymes such as CYP7A1 and CYP8B1. And the first step of the alternative pathway is when the classical synthetic pathway is restricted during disease initiated by CYP27A1. Different from the primary BAs -CA and CDCA synthesized in the human body, CA and MCAs (mainly β-MCA) were synthesized in rodents (Wahlström et al., 2016). They are bound to taurine or glycine and then transported by transport proteins such as BSEP and MRP2, and then form micelles with cholesterol, phospholipids, and other substances, and stored in the gallbladder in the form of bile. After ingestion of food by the body, BAs are secreted into the duodenum under the stimulation of cholecystokinin to perform the physiological functions of emulsification and absorption of lipids. In the intestine, microorganisms are involved in the dissociation, dehydrogenation, and dehydroxylation of BAs. Firstly, the degradation of conjugated BA was completed by hydrolysis of the C24 N-acyl amide bond catalyzed by BSH. Afterward, the formation of secondary BA by dehydroxylation of 7α –dehydroxylated (Sivamaruthi et al., 2020). And about 95% of BAs are reabsorbed in the terminal ileum, and which secreted into the portal vein through basolateral bile acid transporters MRP2, etc., and then, circulated to the liver, after which it’s absorbed by NTCP and OATP1 into the hepatocytes (Jia et al., 2018). It was worth mentioning that MRP4 etc, also provides another alternative way for BAs to enter the systemic circulation. Only a small fraction of BAs that cannot be absorbed is excreted from the body. And this portion maintains a dynamic equilibrium with BA biosynthesis to constitute the BAs steady state in the organism. With prolonged HFD administration, the mRNA expression levels of BSEP, MRP2, NTCP, and other transporter proteins in liver tissues were decreased, which aggravates the burden on the liver and aids the further development of the disease. In contrast, treatment with RPMP ethanol extract increased the mRNA expression level of the transporter protein and alleviated the encounter of the liver when the organism was exposed to a long-term HFD management situation.

In fact, in addition to the physiological functions of emulsification and absorption exhibited after meals, BAs are a multi-effect signaling molecule involved in various physiological activities such as energy metabolism of the body–which own biosynthesis was regulated by negative feedback in the organism. FXR plays an important role in BAs, glucose, and lipid metabolism. FXR/SHP double knockout mice exhibited cholestasis and liver injury at 3 weeks of age, while a significant reduction in CYP27A1 gene expression was observed (Anakk et al., 2011). Typically, the atypical nuclear receptor SHP, which lacks a DNA-binding domain, was thought to work in concert with FXR to maintain bile homeostasis–negatively regulating CYP8B1 and CYP7A1 transcript levels by inhibiting the expression of LRH-1 and HNF-4α (Lee et al., 2000; Lee and Moore, 2002; Chiang, 2009). However, the regulatory role of SHP in fatty liver disease remains controversial. SHP signals were up-regulated in the early stages of NAFLD development. However, as the disease progresses, inflammation, liver damage, and fibrosis will trigger signals that inhibit SHP expression (Benet et al., 2015). And which seems to explain the reduced SHP expression level in mouse liver under HFD induction. In addition, the mechanism of triglyceride-lowering by natural or synthetic FXR agonists may also inhibit SREBP-1c expression through the synergistic effect of SHP (Watanabe et al., 2004). However, FXR expression in the liver and intestine may have opposite effects, and inhibit intestinal FXR signaling ameliorates HFD-induced steatosis and obesity (Li et al., 2013; Sun et al., 2019). Administration of FXR-selective agonists to the liver- and intestine-specific FXR-null models mice showed that short-term inhibition of hepatic CYP7A1 gene expression requires activation of FXR in the intestine (Kim et al., 2007). Furthermore, activation of FXR in ileal cells induces the expression of FGF15, while in hepatocytes, the binding of FGF15 to FGFR4 directly inhibits the expression of CYP7A1, and thus it completes the regulation of BAs biosynthesis (Arab et al., 2017). CDCA is known to be the most effective endogenous agonist for FXR, and CDCA levels were significantly elevated under HFD management. And, higher levels of TαMCA and TβMCA have been suggested as endogenous antagonists of FXR were used to explain the phenomenon of FXR-dependent gene low expression in the ileum of GF mice (Sayin et al., 2013). In contrast, it was shown that IM mitigated the repression of the ileal FXR gene and thus affected the metabolism of BAs in the body by reducing the level of T-β-MCA (Sayin et al., 2013).

**FIGURE 13:**
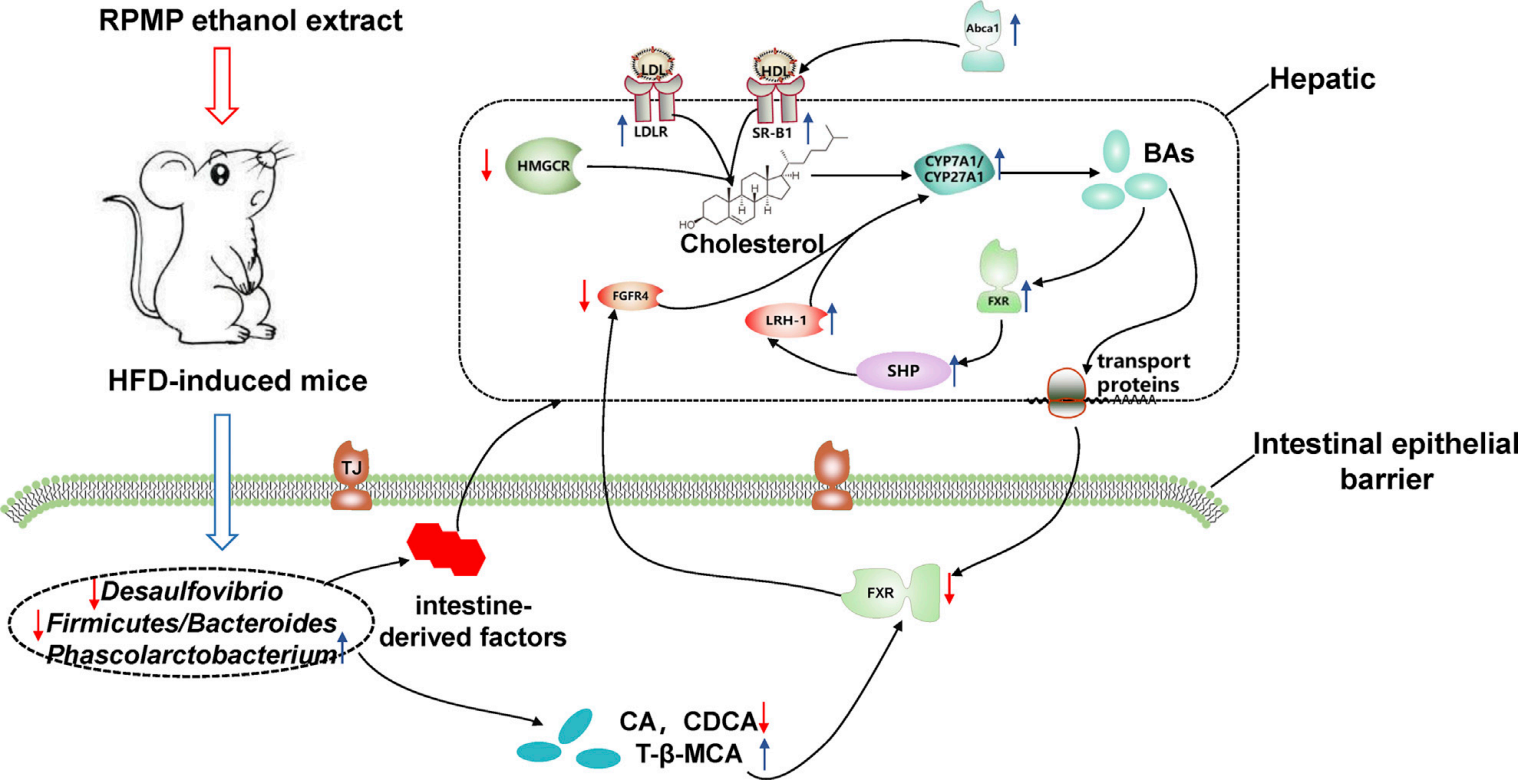
Potential mechanism of action of RPMP alcoholic extracts. The blue arrow showed that the RPMP ethanol extract increased the expression, the red arrow showed that the RPMP ethanol extract decreased the expression.

To sum up the above, during the development of NAFLD in HFD-induced mice, alterations in IM composition resulted in disproportionate species abundance and the production of intestine-derived factors exacerbate the disruption of intestinal barrier function and increase the risk of direct exposure of the liver to the intestinal tract. Moreover, the long-term intake of a high-fat and high-cholesterol diet causes disorders of lipid metabolism in the body, which provides a suitable living environment for bacteria, and undoubtedly forms a vicious circle with IM disorders to accelerate the disorder of the body’s micro-ecological environment, contributing to the development of NAFLD. At the same time, the disordered IM structure further exacerbates the development of NAFLD by affecting cholesterol metabolism. In contrast, treatment with RPMP ethanol extract remodeled the HFD-induced structural dysregulation of IM in mice, which also facilitated the restoration of intestinal barrier function and reduced the risk of liver exposure to intestinal-derived factors. More importantly, the remodeling of IM structure influenced the metabolism of BAs in the intestine, and BAs, as signaling molecules, further feedback regulated the cholesterol metabolism and maintained the body cholesterol homeostasis (Figure 13).

Since the doses used in this study were converted based on the doses administered between animals and humans, and the dose levels obtained based on this may result in artefacts in the models used (Heinrich et al., 2020). Thus, it was recommended to study more therapeutically relevant dose levels as a next step.

## Conclusion

In conclusion, RPMP ethanol extract can effectively improve the abnormal lipid metabolism and hepatic lipid accumulation caused by HFD. IM plays an important role in this process. Firstly, the imbalance of IM structure caused by HFD was improved by RPMP ethanol extract, and the function of the intestinal barrier affected by IM was restored, which showed the increase of expression of JAM, ZO-1, and Occludin genes. Secondly, the remodeled IM affected the metabolism of BAs, specifically, the increased T-β-MCA levels inhibited the expression of intestinal FXR genes, which in turn upregulated the expression of CYP7A1 genes through FGFR4 genes, accelerating cholesterol metabolism in the body and maintaining cholesterol homeostasis (Figure 13). And this process was also confirmed in the Chinese pharmacopoeia of the RPMP melting turbidity and reducing lipid efficacy described (Commission, 2020). Of course, we should also bear in mind that a good lifestyle is fundamental to prevent problems before they occur. However, there were some limitations to our study. Firstly, it was difficult to dynamically measure the effect of RPMP ethanol extract on the IM structure during the development of NAFLD due to the small sample size and lack of IM detection in mice at different stages. Secondly, our study lacked the detection of serum BAs profile, while the composition structure of serum BAs could provide a more accurate understanding of the metabolism of host BAs. Finally, because of the numerous components in RPMP, prediction by network pharmacology complemented by corresponding validation experiments will be our next focus to explore the active compounds in RPMP.

## Data Availability

The data presented in the study are deposited in the Sequence Read Archive (SRA) portal of NCBI repository (https://www.ncbi.nlm.nih.gov/sra/), accession number PRJNA769303.

## Abbreviations

NAFLD, non-alcoholic fatty liver disease; NASH, non-alcoholic steatohepatitis; RPMP, *Polygoni Multiflori Radix Praeparata*; ND, normal diet; HFD, high-fat diet; TSG, 2,3,5,4′-tetrahydroxystilbene-2-O-β-d-glucoside; HPLC, high performance liquid chromatography; TC, total cholesterol; TG, triglyceride; LDL-C, low-density lipoprotein cholesterol; HDL-C, high-density lipoprotein cholesterol; ALT, alanine transaminase; AST, aspartate transaminase; IM, intestinal microbial; BAs, bile acids; IR, insulin resistance
